# Probiotic Bacteria: A Promising Tool in Cancer Prevention and Therapy

**DOI:** 10.1007/s00284-019-01679-8

**Published:** 2019-04-04

**Authors:** Agata Górska, Dawid Przystupski, Magdalena J. Niemczura, Julita Kulbacka

**Affiliations:** 1 Department of Biological Sciences, Institute of Experimental Biology, University of Wrocław, Kanonia 6/8, 50-328 Wrocław, Poland; 2 Faculty of Medicine, Wroclaw Medical University, J. Mikulicza-Radeckiego 5, 50-345 Wrocław, Poland; 3 Department of Molecular and Cellular Biology, Wroclaw Medical University, Borowska 211A, 50-556 Wrocław, Poland

## Abstract

Gut microbiota is widely considered to be one of the most important components to maintain balanced homeostasis. Looking forward, probiotic bacteria have been shown to play a significant role in immunomodulation and display antitumour properties. Bacterial strains could be responsible for detection and degradation of potential carcinogens and production of short-chain fatty acids, which affect cell death and proliferation and are known as signaling molecules in the immune system. Lactic acid bacteria present in the gut has been shown to have a role in regression of carcinogenesis due to their influence on immunomodulation, which can stand as a proof of interaction between bacterial metabolites and immune and epithelial cells. Probiotic bacteria have the ability to both increase and decrease the production of anti-inflammatory cytokines which play an important role in prevention of carcinogenesis. They are also capable of activating phagocytes in order to eliminate early-stage cancer cells. Application of heat-killed probiotic bacteria coupled with radiation had a positive influence on enhancing immunological recognition of cancer cells. In the absence of active microbiota, murine immunity to carcinogens has been decreased. There are numerous cohort studies showing the correlation between ingestion of dairy products and the risk of colon and colorectal cancer. An idea of using probiotic bacteria as vectors to administer drugs has emerged lately as several papers presenting successful results have been revealed. Within the next few years, probiotic bacteria as well as gut microbiota are likely to become an important component in cancer prevention and treatment.

## Introduction

Cancer is considered as one of the most significant causes of death. The treatment of tumors has received much attention in the last years; however, the number of people suffering neoplastic syndrome is still increasing. Thus, researchers are trying to face this process searching for innovative therapies and prophylaxis. Despite the fact that cancer risk indisputably depends on genetic factors, immunological condition of the organism plays a considerable role in it, that being closely associated with probiotic bacteria and commensal bacterial flora presented mainly in the digestive tract. Probiotic strains, inter alia *Bifidobacterium*, or *Lactobacillus*, widely present in commonly consumed fermented milk products, are known to have various beneficial effects on health. To date, there is a plethora of studies investigating the correlation between intestinal microbiota and carcinogenesis which have been evaluated in this article. A growing body of research has been analyzed and reviewed for the potential application of probiotics strains in prevention and treatment of cancer.

### Probiotics and Cancer

Goldin and Gorbach [1] were among the first to demonstrate the association between a diet enriched with Lactobacillus and a reduced incidence of colon cancer (40% vs. 77% in controls). Probiotics have been gaining much attention due to their ability to modulate cancer cell’s proliferation and apoptosis, investigated both in vitro (Table 1) and in vivo (Table 2). Potential application of these properties in novel therapy could potentially be alternative to more invasive treatment such as chemotherapy or radiotherapy.

**Table 1:** General effects of probiotics on cancer cells in vitro

Probiotic strain/details of experiment	Cell line	Effect	References
*Bifidobacterium adolescentis* SPM0212 /cell free supernatant used/	Caco-2, HT-29, SW480	↓ Cell proliferation	[2]
*Enterococcus faecium* RM11 *Lactobacillus fermentum* RM28	Caco-2	Cell proliferation: ↓ 21% ↓ 23%	[3]
*Lactobacillus rhamnosus* GG *Bifidobacterium lactis* Bb12	Caco-2	↑ Apoptosis	[4]
*Bacillus polyfermenticus*	HT-29, DLD-1, Caco-2	↓ Cell proliferation N/E on apoptosis	[5]
*Bacillus polyfermenticus* /AOM stimulation/	NMC460	↓ Cell colony formation in cancer cells (N/E on normal colonocytes)	[5]
*Lactobacillus paracasei* IMPC2.1 *Lactobacillus rhamnosus* GG /heat killed/	DLD-1	↓ Cell proliferation Induction of apoptosis	[6]
*Pediococcus pentosaceus* FP3, *Lactobacillus salivarius* FP25/FP35, Enterococcus faecium FP51	Caco-2	↓ Cell proliferation Activation of apoptosis	[7]
*Lactobacillus plantarum* A7 *Lactobacillus rhamnosus* GG /heat killed, cell free supernatant used/	Caco-2, HT-29	↓ Cell proliferation	[8]
*Clostridium butyricum* ATCC *Bacillus subtilis* ATCC 9398	HCT116, SW1116, Caco-2	↓ Cell proliferation	[9]
*Bacillus polyfermenticus* KU3	LoVo, HT-29, AGS	>90% ↓ Cell proliferation	[10]
*Lactococcus lactis* NK34	HT-29, LoVo, AGS	>80% ↓ Cell proliferation	[11]
*Lactobacillus casei* ATCC 393	HT29 and CT26	Induction of apoptosis	[12]
*Lactobacillus pentosus* B281 *Lactobacillus plantarum* B282 /cell free supernatant used/	Caco-2 and HT-29	↓ Cell proliferation Cell cycle arrest (G1)	[13]

↓ Decrease; ↑ increase; *N/E* no effect. Human colonic cancer cells: Caco-2, HT-29, SW1116, HCT116, SW480, DLD-1, LoVo, Human colonic epithelial cells: NMC460. Human gastric adenocarcinoma cells: AGS *Mus musculus* colon carcinoma cells: CT26

**Table 2:** General effects of probiotics on tumor-bearing or tumor-induced animal models in vivo

Probiotic strain	Model	Induction	Treatment	Result	References
*Lactobacillus acidophilus*, *Lactobacillus casei* *Lactobacillus lactis* biovar *diacetylactis* DRC-1	Rat	DMH	40 weeks	↓ TI ↓ TV ↓ TM	[14]
*Bifidobacterium lactis* KCTC 5727	SPF C57BL rat	–	19 weeks	↓ TI ↓ TV	[15]
*Bacillus polyfermenticus*	CD-1 mice	DLD-1 cells injection	20 weeks (injection)	↓ TI ↓ TV	[5]
*VSL#3* (Probiotics mixture)	SD rats	TNBS	10 weeks	None of the animals developed CRC	[16]
*Lactobacillus rhamnosus* GG MTCC #1408 *Lactobacillus acidophilus* NCDC #1	SD rats	DMH	19 weeks^a^	↓ TI ↓ TM	[17]
*Lactobacillus plantarum*	BALB/c mice	AOM, DSS	Nanosized/ Live bacteria 4 weeks	↓ TI cell cycle arrest Induction of apoptosis	[18]
*Lactobacillus plantarum*	BALB/c mice	CT26 cells injection	14 weeks	↓ TV Induction of necrosis	[19]
VSL#3 (Probiotics mixture)	C57BL/6 mice	DSS	^a^	↓ TI ↓ dysplasia	[20]
*Lactobacillus plantarum* (AdF10) *Lactobacillus rhamnosus* GG	SD rats	DMH 4 weeks	One of strains 12 weeks	↓ TI ↓ TV ↓ TM	[21]
*Lactobacillus salivarius* Ren	F344 rats	DMH 10 weeks	2 weeks^a^	↓ TI	[22]
*Lactobacillus acidophilus* *Bifidobacteria bifidum Bifidobacteria infantum*	SD rats	antibiotics DMH	23 weeks	↓ TI ↓ TV	[23]
*Lactobacillus rhamnosus* GG CGMCC 1.2134	SD rats	DMH 10 weeks	25 weeks	↓ TI ↓ TV ↓ TM Induction of apoptosis	[24]
*Pediococcus pentosaceus* GS4	Swiss albino mice	AOM	4 weeks	↓ TP Induction of apoptosis	[25]
*Lactobacillus casei* BL23	C57BL/6 mice	DMH	10 weeks	↓ TI	[26]

^a^Before and until the end of experiment

↓ Decrease, *TI* tumor incidence, *TV* tumor volume, *TM* tumor multiplicity, *TP* tumor progression, *AOM* azoxymethane, *CRC* colorectal cancer, *DMH* 1,2 dimethylhydrazine dihydrochloride, *DSS* dextran sulfate sodium, *TNBS* trinitrobenzene sulfonic acid, *SD rat* Sprague–Dawley rat

### Mechanisms of Action

A specific mechanism associated with antitumor properties of probiotics remains unclear. Gut microbiota is engaged in a variety of pathways, which are considered to play a central role in that process. Primarily, probiotic bacteria play an essential role in the preservation of homeostasis, maintaining sustainable physicochemical conditions in the colon. Reduced pH caused inter alia by the excessive presence of bile acids in feces may be a direct cytotoxic factor affecting colonic epithelium leading to colon carcinogenesis [27, 28]. Regarding their involvement in the modulation of pH and bile acid profile, probiotic bacteria such as *L. acidophilus* and *B. bifidum* have been demonstrated to be a promising tool in cancer prevention [27, 29, 30].

Probiotic strains are also responsible for maintaining the balance between the quantity of other participants of natural intestinal microflora and their metabolic activity. Putrefactive bacteria, such as *Escherichia coli* and *Clostridium perfringens* naturally present in the gut, has been proven to be involved in production of carcinogenic compounds using enzymes like b-glucuronidase, azoreductase, and nitroreductase. Some preliminary research conducted by Goldin and Gorbach in the late 1970s have proven consumption of milk fermentation products to have a beneficial effect on the increase in the number of *L. acidophilus* in rat’s gut, which subsequently resulted in a reduction of putrefactive bacteria and decrease in the level of harmful enzymes [31]. Several subsequent studies confirmed the positive influence of the probiotic strains on the activity of bacterial enzymes implicated in the tumor genesis both in humans [32, 33] and rodents [1, 31, 34–38]. It is worth noting that there is considerable ambiguity among the gathered data; nevertheless, results concerning glucuronidase and nitroreductase are in general consistent. However, whether these processes affect cancer rates in humans is yet to be investigated [39].

Another cancer-preventing strategy involving probiotic bacteria, chiefly *Lactobacillus* and *Bifidobacillus* strains, could be linked to the binding and degradation of potential carcinogens. Mutagenic compounds associated with the increased risk of colon cancer are commonly found in unhealthy food, especially fried meat. Ingestion of *Lactobacillus* strain by human volunteers alleviated the mutagenic effect of diet rich in cooked meat, which resulted in a decreased urinary and fecal excretion of heterocyclic aromatic amines (HAAs) [40, 41]. Supplementation with dietary lactic acid bacteria has shown to downregulate the uptake of 3-amino-1-methyl-5H-pyrido (4,3-β) indole (Trp-P-2) and its metabolites in mice [42]. Furthermore, many in vitro studies have been conducted, demonstrating the ability of different probiotics strains to either bind [43–51] or metabolize [43, 47, 49] mutagenic compounds such as HAAs [44–47, 49, 50], nitrosamines [43, 49], aflatoxin B1 [48], and others: mycotoxins, polycyclic aromatic hydrocarbons (PAHs), and phthalic acid esters (PAEs) [52]. In some cases investigation revealed the correlation of these properties with the reduction of mutagenic activities presented by the aforementioned compounds [43, 45–47, 50, 53]. It is worth highlighting that the substantial part of a current knowledge on the phenomenon discussed above is largely based on in vitro studies. All these results should be interpreted with caution, according to the variations of factors such as pH, occurring at in vivo conditions, which can potentially alter the efficiency of binding or degradation of the mutagens [52].

Many beneficial compounds produced and metabolized by gut microbiota have been demonstrated to play an essential role in maintaining homeostasis and suppressing carcinogenesis. Specific population of gut microbiota are dedicated to production of short-chain fatty acids (SCFAs) such as acetate, propionate, and butyrate as a result of the fermentation of fiber-rich prebiotics. Except for their principal function as an energy source, SCFA have also been proven to act as signaling molecules affecting the immune system, cell death, and proliferation [54] as well as the intestinal hormone production and lipogenesis, which explains their crucial role in epithelial integrity maintenance [55].

Although lactic acid bacteria are not directly involved in SCFA production, certain probiotic strains of *Bifidobacteria* and *Lactobacilli* can modulate the gut microbiota composition and consequently affect the production of SCFA [56]. Butyrate, produced by species belonging to the *Firmicutes* families (*Ruminococcaceae*, *Lachnospiraceae*, and *Clostridiaceae*) [55] has been proven to promote apoptosis and inhibit proliferation in cancer cells cultured in vitro [57] and remains the most investigated of SCFAs. Colorectal cancer is strongly correlated with decreased levels of SCFA and SCFA-producing bacteria dysbiosis [58]. Administration of bacterial strain *Butyrivibrio fibrisolvens* MDT-1, (known for their high production of butyrate) in mouse model of colon cancer, inhibited progression of tumor development, affecting also the reduction of β-glucuronidase and increasing the immune response [59].

More recent evidence suggests modulation of SCFA-producing bacteria by dietary intervention with fermentable fibers as a possible colorectal cancer treatment. A more recent study on mice demonstrated amelioration of polyposis in CRC (colorectal cancer) after increasing SCFA-producing bacteria after introduction of probiotic diet. Previously investigated application of synbiotic combination of *B. lactis* and resistant starch in rat-azoxymethane model has been proven to protect against the development of CRC, which was correlated with increased SCFA production [60]. Interestingly, neither *B. lactis* nor prebiotic were sufficient to achieve that effect alone. This and some previous assays suggest that prebiotic activity of fiber-enriched diet, projecting on the level of beneficial bacteria, is promising strategy to prevent CRC.

Lactic acid bacteria have been receiving much attention due to its contribution to immunomodulation correlated with either suppression or regression of carcinogenesis. This phenomenon is the result of multidimensional activity involving interaction between the bacteria or their metabolites with the immune and epithelial cells [9, 19, 61–63]. Consequentially, probiotic strains have the ability to both increase and decrease the production of anti-inflammatory cytokines as well as modulate secretion of prostaglandins, which altogether projects on suppression of carcinogenesis. Another strategy involves activation of phagocytes by certain probiotic strains, leading to direct elimination of early-stage cancer cells [58, 62]. For a detailed review, see a comprehensive elaboration recently published in *Nature* summarizing the mechanisms engaging microbiota in immune homeostasis and disease [64].

It has been demonstrated that some probiotics strains of Lactobacilli have been proven to suppress gastric-cancer-related *H. pylori* infections [65–67]. Another study conducted on patients with persistent human papillomavirus virus (HPV) showed an enhanced clearance of HPV and cervical cancer precursors after daily consumption of probiotics for 6 months [68].

### Probiotics in Cancer Therapy

In recent years, there has been growing interest in the possible application of probiotics as a part of combination therapy with conventional treatment of cancer. An early but controlled and comparative study on 223 patients carried out in 1993 showed that combination therapy including radiation and treatment with heat-killed *L. casei* strains (LC9018) and improved the induction of immune response mechanisms against cancer cells thereby enhancing tumor regression in patients with carcinoma of the uterine cervix [69]. Research on azoxymethane-induced CRC mice model treated by the probiotic mix composed of seven different strains of lactobacilli, bifidobacteria, and streptococcus demonstrated suppression of colon carcinogenesis due to modulation of mucosal CD4+ T polarization and changes in the gene expression [70]. Furthermore, latest experiment investigating the effects of *B*.* infantis* administration in CRC rat model demonstrated a considerable attenuation of chemotherapy-induced intestinal mucositis correlated with decreased level on proinflammatory cytokines (IL-6, IL-1β, TNF-α) and increased CD4+ CD25+ Foxp3+ T regulatory cell response [71].

Over and above that, two seminal papers published in *Science* highlighted the significant role played by gut microbiota in the immune response to cancer treatment. Disruption of the microbiota in mice was made evident by a decreased immune response and thereby tumor resistance for either cyclophosphamide [72] or oxaliplatin therapy [73]. As a result of these findings, probiotic bacteria have been gaining traction as a crucial component in successful cancer immunotherapy [63, 74–76].

The most recent experiments on mice have illustrated the key role of gut microbiota (*Bacteroides* and *Bifidobacterium*) in anti-PD-L1 (Programmed death-ligand 1) and anti-CTLA-4 (cytotoxic T lymphocyte-associated protein 4) therapies [77, 78]. Immunomodulatory effect was exhibited in intensified activation of dendritic cells and also promotion of antitumor T cell response. Essentially, Sivan et al. [77] observed a similar improvement of tumor control as a result of *Bifidobacterium* treatment alone compared to anti–PD-L1 therapy, whereas combination of both strategies was sufficient to nearly eliminate tumor outgrowth. These groundbreaking results indicate that administration of probiotics appears to be a promising strategy in maximizing the efficiency of cancer immunotherapy.

### Cohort Studies

Several cohort studies have revealed the correlation between the consumption of dairy products and the risk of colon cancer. Some of these findings appear useful in drawing conclusions concerning the role of probiotic bacteria in carcinogenesis, taking into account certain groups of previously investigated dairy products such as fermented milk products with a special emphasis on yogurt. There is still considerable ambiguity among studies, summarized in Table 3.

**Table 3:** Cohort studies investigating the correlation between the consumption of dairy products and the cancer risk

Study	Country	Years	No. of participants	Products	Result
Järvinen (2001) [79]	Finland	1966–1972	9959	Milk and dairy products	I/A^a^
van’t Veer (1994) [80]	United states	1986–1989	120,852	Fermented dairy products	Slight I/A
Kearney (1996) [81]	United states	1986–1992	47,935	Milk and fermented dairy products	N/S/A
Pietinen (1999) [82]	Finland	End 1993	27,111	Milk and dairy products	I/A
Lin (2005) [83]	United States	1993	39,876	Milk fermented and unfermented dairy products	N/S/A
Larsson (2006) [84]	Sweden	1997–2004	45,306	Dairy products	I/A

^a^Without specific effects of fermented milk

*I/A* inversed associations between intake and cancer risk *N/S/A* no significant associations

In contrast to that uncertainty, a recent study conducted in 2012 produced a meta-analysis including nineteen cohort studies which demonstrated an association between consumption of dairy products (except cheese) and a decreased colorectal cancer risk [85]. Another noteworthy approach investigating the influence of dairy products on post-diagnostic CRC survival clearly indicates positive correlation between the high dairy intake and the lower risk of death [86].

A key problem with the majority of the cohort studies mentioned above is that they covered general dairy intake, including high-fat components such as cream and cheese, suspected of carcinogenic properties due to their ability to increase bile acid levels in the colon [85, 87]. Moreover, research tends to focus on anticancer compounds such as calcium or vitamin D, without paying special attention to probiotics. Therefore, the first innovative cohort study conducted in 2011 by Pala et al. [88] on 45,241 subjects proved a significant association between single probiotic-rich product intake (yogurt) and decreased colon cancer risk. Similar approaches should be conducted on large cohorts, investigating probiotics’ intake from natural sources (such as yogurt and other fermented dairy products) as well as supplements, in order to reveal their effect on cancer risk.

### Probiotics in Treatment and Prophylaxis

**Fig. 1:**
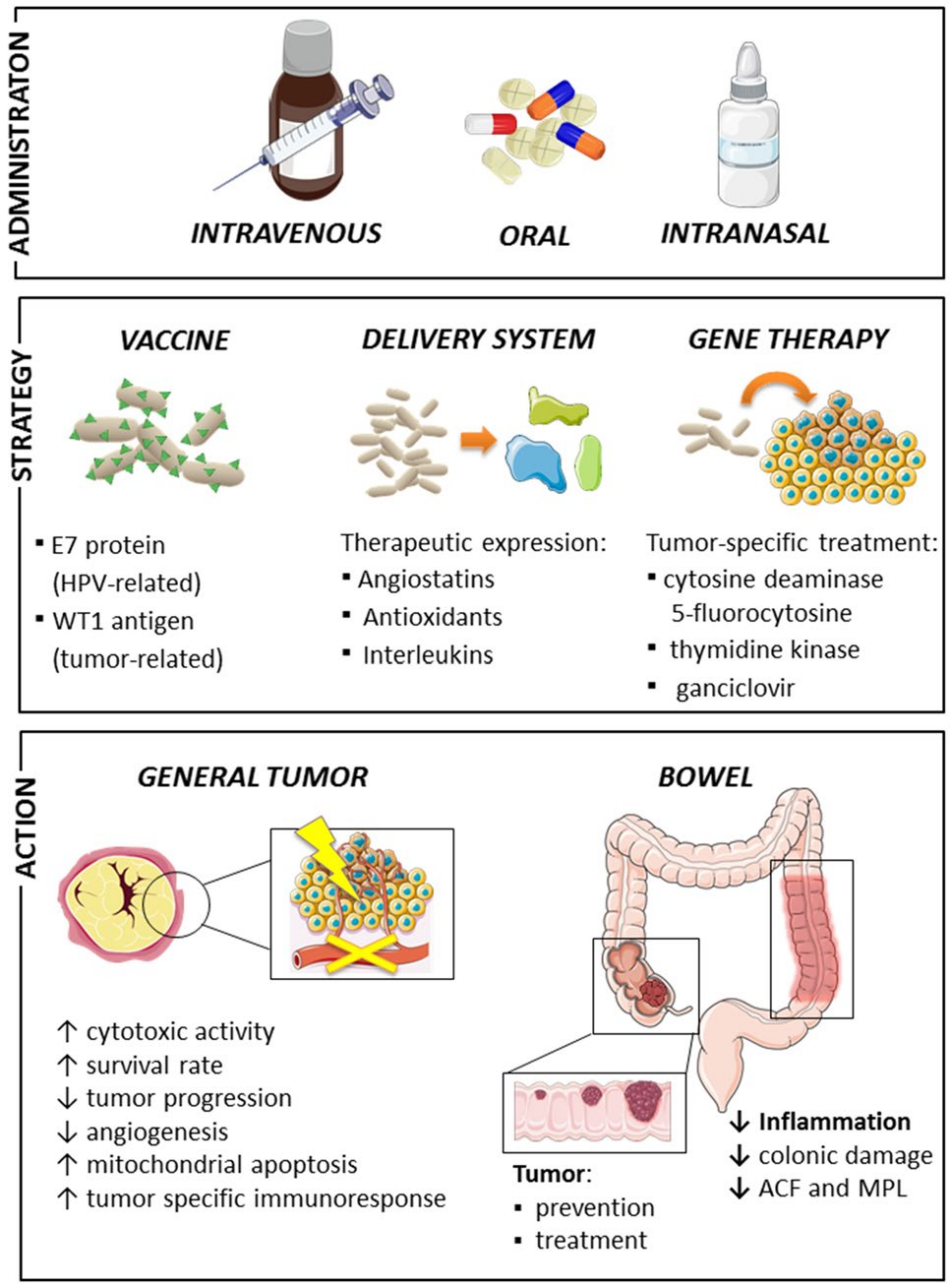
Summary of the possible applications of probiotic bacteria in the treatment and prevention of cancer. Figure summaries most significant findings from studies in vitro and in vivo mentioned in text [89–114]. This figure was prepared using Servier Medical Art, available from www.servier.com/Powerpoint-image-bank. Legend: *downwards arrow* decrease, *upwards arrow* increase *ACF* aberrant crypt foci, *MPL* multiple plaque lesions

Utilization of the recombinant probiotic strains as a delivery system for various therapeutic molecules such as drugs, as well as cytokines, enzymes, or even DNA [89, 90] is quite recent and exceptional idea that could be successfully applied for colorectal cancer treatment (Fig. 1). Probiotic bacteria are indispensable as vectors due to their wide range of tolerance to the environment of gastrointestinal tract co-occurring with their natural capability of colonizing the mucosal surface followed by prolonged residence maintaining their protective properties [91]. The innovative concept of a “bio drug” relies on oral administration of genetically modified probiotics allowing a direct delivery of the therapeutic components to the intestinal mucosa. Regarding low costs, simple technology, and procedure of the treatment, this strategy has a great potential to be widely used in prevention and treatment of various disorders.

In several independent studies on rodents, intragastric application of recombinant strains of *Lactobacillus lactis* expressing anti-inflammatory compounds (cytokines, IL-10, human interferon-beta, or antioxidants) has been shown to ameliorate the intestinal inflammation and demonstrated cytoprotective effect [92–94]. In another approach, application of *Lactococcus lactis* expressing catalase has been proven to decrease the production of reactive oxygen species (ROS) such as H_2_O_2_, reducing colonic damage, and inflammation, consequently projecting on tumor invasion and proliferation [95].

More recent study investigating multiple strategies of inhibition of the inflammatory-related carcinogenesis with different combination of probiotic vectors expressing antioxidant enzymes (catalase, superoxide dismutase) or IL-10 (produced as cDNA or in expression system inducible by stress—SICE) has shown these strains as agents causing significant changes of the immune response as well as pre-neoplastic lesions or even causing the entire inhibition of tumor development [96] (for details see Table 4).

**Table 4:** Comparison of the strategies using the probiotic strains in cancer prevention and treatment

Probiotic strains	Model	Treatment	Effect	References
Probiotic vaccination				
*Lactococcus lactis*	C57BL/6 mice → *Intranasal*	E7 protein displayed	↑ Antitumor effect of following Ad-CRT-E7 treatment	[104]
*Lactococcus lactis*	C57BL/6 mice → *Intranasal*	E7 protein displayed	HPV-16 E7-specific immune response	[103]
*Bifidobacterium longum*	C57BL/6N mice *inj/w* C1498-WT1 → *Oral*	WT1 displayed	↓ WT1-expressing Tumor growth ↑ Survival rate ↑ Tumor infiltration of CD4+ T and CD8+ T ↑ Cytotoxic activity	[96]
Mitigation of inflammation				
*Streptococcus thermophilus* *Lactococcus lactis*	BALB/c mice (DMH)-I CRC → *Oral*	Antioxidant enzymes (catalase, superoxide dismutase), IL-10; Groups: IL-10 (SICE) IL-10 (cDNA) antioxidants, mix	All groups: ↓ Tumor incidence ↓ ACF and MPL ↓ MCP-1 ↑ IL-10/TNFα Groups: IL 10 (SICE), antioxidants and mix: no tumor Mix: ↓↓ ACF and MPL ↓↓ MCP-1 ↑↑ IL-10/TNFα	[96]
*Lactococcus lactis*	DSS-induced mice → *Intragastric*	IL-10	No tumor ↓ Colonic damage ↓ Inflammation	[92]
*Lactococcus lactis*	BALB/c mice (DMH)-I CRC → *Oral*	Catalase	↓ Colonic damage ↓ Inflammation ↓ Tumor incidence ↓ Tumor progression	[95]
Drug delivery				
*Bifidobacterium longum*	BALB/c mice *inj/w* CT24 → *Oral* or *injection*	Tumstatin	Antitumor effect	[111]
*Lactococcus lactis*	Rats (DMH)-I CRC → *Oral*	Endostatin	↑ Survival rate N/E on complete cure	[115]
*Bifidobacterium longum*	C57BL/6 mice *inj/w* Lewis lung cancer and B16-F10 → *Oral*	Endostatin or endostatin + selenium	Endostatin group: ↓ Tumor progression ↑ Survival time Endostatin ± selenium: ↓↓ Tumor progression ↑ Activity of NK, T cells and ↑ Activity of IL-2 and TNF-a i	[112]
Gene therapy				
*Bifidobacterium infantis*	Melanoma B16-F10 cells → Supernatant fluid	Cytosine deaminase/5-fluorocytosine	↑ Morphological damage ↓ Growth	[116]
	C57BL/6 Mice, inj/w B16-F10 cells → *Injection*	Cytosine deaminase/5-fluorocytosine	Antitumor effect	
*Bifidobacterium infantis*	BALB/c Mice and cell lines: Colo320, MKN-45, SSMC-7721, MDA-MB-231 → *Injection*	Thymidine kinase (BF-rTK) Ganciclovir (GCV)	↑ Mitochondrial apoptosis ↓ Inflammation ↓ TNFα	[113]

→ Administration, *inj/w* injected with, ↓ decrease, ↑ increase, *N/E* no effect. Cell lines: human: Colo320—colon adenocarcinoma MKN-45—gastric cancer, MDA-MB-231—breast cancer, SSMC-7721—liver cancer. Mouse: B16-F10—skin melanoma, CT24*—*colorectal cancer, C1498-WT1—leukemia

*ACF and MPL* pre-neoplastic lesion: aberrant crypt foci and multiple plaque lesions, *CRC* colorectal cancer, *DMH-I* 2-dimethylhydrazine induced, *DSS* dextran sulfate sodium, *HO-1* Heme oxygenase-1, *IL-10* interleukin 10, *MCP-1* monocyte chemoattractant protein 1 (cytokine), *MT* mammary tumor, *S–D* Sprague–Dawley (rats), *TNFα* tumor necrosis factor

A plethora of studies reported potential application of the probiotic expression systems as vaccines, demonstrating stimulation of the adaptive immune system response against the pathogens [97–99]. A number of experiments investigating application of genetically engineered probiotics expressing human papillomavirus E7 oncoprotein or the treatment of cervical cancer have shown that in contrast to the traditional polyvalent vaccines, which have preventive properties only on the development of the disease, “probiotic vaccination” has been demonstrated to have both protective (stimulating immunological response) and therapeutic effects (tumor regression) [100–103]. Pre-immunization with E7-displaying lactococci significantly enhanced the antitumor effect of a following treatment with adenovirus [104].

Studies on TC-1 tumor murine model have shown that therapeutic effect can be enhanced by co-administration of *Lactobacillus lactis* capable of expressing oncoprotein E7 and immunostimulatory compounds, such as interleukin-12 [96, 101, 102]. Prophylactic administration of the vaccine in healthy individuals conferred to resistance to subsequent administration of lethal levels of tumor cell line TC-1, even after the second induction, resulting in 80 [102] to 100% [101] survival rate. Treatment of tumor-bearing mice with recombined probiotic caused regression of palpable tumors, correlated with the increased antitumor cytotoxic T lymphocyte (CTL) immunoresponse [101, 102].

Most recent evidence proposes the utilization of probiotics in the delivery of tumor-associated antigens (TAAs) as an orally administrated vaccine, based on a recently reported prosperous approach with *Bifidobacterium* expressing Wilms’ tumor 1 (WT1) protein [105].

Occurrence of hypoxic and neurotic areas among solid cancer tissues gives rise to the opportunity of utilization of a specific tendency of certain probiotic strains for selective localization and proliferation in anaerobic environment [106–109]. This phenomenon was further investigated in rodents, leading to the evaluation of direct anticancer treatment using *Bifidobacteria* as a delivery vehicle for specific drugs such as cytosine deaminase [110] or angiostatins [111, 112] or even in gene therapy [113].

The most important limitation of abovementioned strategies lies in the fact that genes for antibiotic resistance, commonly used as selective marker in the procedure of cloning, could be potentially transferred to resident intestinal microbiota by probiotic delivery vectors. Finding an alternative, secure selection marker for cloning in therapeutic strains still remains a challenging area in this field [114].

## Conclusions

This paper has given an account of the role played by gut microbiota in cancer prevention and treatment. It is noteworthy that until now most of these innovative methods mentioned above have only been investigated in animal models. Clinical tests of this strategy are expected to raise a possibility of utilizing probiotic bacteria as comprehensive drug-delivery vectors for non-invasive cancer treatment in humans. Taken together, a growing body of literature had highlighted a role of probiotic balance in maintenance of widely understood homeostasis, projecting on successful cancer therapy. The evidence from latest studies points towards the idea of possible implementation of probiotics in cutting-edge cancer therapies. Future investigations on the current topic are therefore necessary in order to validate these findings and establish therapeutic strategies. This could conceivably lead to a breakthrough in various fields of medicine not only supporting immunotherapy in cancer treatment or elaboration and production of an innovative vaccines, but also improving drug delivery in other bowel diseases while preventing and mitigating inflammation at the same time.
